# Blood pressure measurement in dental offices and dentists’ cardiovascular risk management: A cross-sectional study

**DOI:** 10.4317/jced.62888

**Published:** 2025-07-01

**Authors:** Johanna Otero, Mario Guerrero, Yamileth Ortiz-Gomez

**Affiliations:** 1 DDS, MPH. Facultad de Odontología, Universidad Santo Tomás, Bucaramanga, Colombia; 2 DDS. Facultad de Odontología, Universidad Santo Tomás, Bucaramanga, Colombia; 3 BSc, PhD. Programa de Doctorado en Salud Pública, Universidad El Bosque, Bogotá, Colombia

## Abstract

**Background:**

Blood pressure measurement (BPM) is a primary test for detecting and managing cardiovascular risk, is an inexpensive strategy and can be performed by non-physician health workers (NPHWs). This study explored the association between the BPM in dental offices and other dentists’ practices related to cardiovascular risk management.

**Material and Methods:**

A cross-sectional study was conducted. A self-administered electronic survey was used, validated by experts with more than 10 years of clinical, teaching and research experience. Simple random sampling was used to select the participants at an anonymized database. The survey was completed by dentists who practice clinically at least part-time in Colombia. Descriptive and multivariate analyses were conducted.

**Results:**

A total of 232 dentists were interviewed. Blood pressure was measured in the dental office by 40.5% of the dentists, with 27.2% using an automatic device. After adjusting for age, education, and support staff, an association was observed between the absence of BPM in dental offices and the following practices: not inquiring about alcohol use, physical inactivity, high cholesterol, obesity, a lower likelihood of measuring glucose in the dental office, not referring patients to a laboratory for glucose measurement, not communicating with medical professionals to arrange patient treatment, and not educating people with obesity about the risk of systemic disease.

**Conclusions:**

BPM in dental offices is limited. Most dentists report inquiring about modifiable cardiovascular risk factors, as well as making referrals, providing counseling, and offering education. However, the absence of BPM in dental offices is associated with the omission of other practices related to cardiovascular risk management. NPHWs like dentists can task sharing cardiovascular risk management.

** Key words:**Blood pressure, hypertension, cardiovascular risk factors, primary prevention, dental office.

## Introduction

Cardiovascular disease (CVD) is the leading cause of death worldwide, except for the 2020 and 2021 pandemic period (1). Analysis of a South American cohort, with a mean follow-up of 10.3 years shows that a significant proportion of CVD and premature deaths could be prevented by controlling cardiometabolic risk factors. For incident cardiovascular events, the largest population attribuTable fractions (PAFs) were due to hypertension (18.7%), which also contributed to 12.0% of cardiovascular deaths (2). Despite widespread knowledge about ways to prevent and treat hypertension, the prevalence of this condition and, more importantly, its cardiovascular complications remain unchanged (3,4).

Due to inadequacies in the prevention, diagnosis, and control of hypertension, efforts have been made to enhance awareness of the condition, among other key objectives (5-7) and to prioritize a comprehensive set of evidence-based interventions to improve cardiovascular health (8-11). As part of adopting global best practices in the prevention and control of CVD, improving hypertension management involves measuring blood pressure with validated devices that ensure accurate readings. This is crucial for the effective implementation and scaling-up of hypertension control programs (8-10).

Recently, Schwalm *et al*., published a review of strategies for the upscaling of established interventions that would be most impactful in reducing the burden of CVD (14). The importance of CVD risk assessment and education through task sharing to non-physician health workers (NPHWs), community health workers or village volunteers, has been emphasized. While the Bangkok Declaration (15) recognizes the fundamental role of dentists in public health, urging them to go beyond traditional oral care and actively engage in preventing and controlling non-communicable diseases. NPHWs like dentists, as part of a in multidisciplinary team, can play an important role in detecting risk factors, such as high blood pressure, and educating patients about cardiovascular health. This study also aligns with World Health Organization recommendations and action plan on oral health (15,16).

However, studies assessing dental practices suggest that dentists are not evaluating the proposed relationship between oral and cardiovascular health (17), and that there is a lack of knowledge regarding the proper management of patients with hypertension (18). Additionally, dentists may not be incorporating this evidence into clinical practice (17). Particularly, dentists’ knowledge and practices regarding blood pressure measurement (BPM) may be deficient or inadequate. This may be attributed to the infrequent use of BPM in practice (19) at best, only one in four dentists measure blood pressure in all patients (17,18,20). In this context, the primary objective of the present study was to investigate the association between BPM in dental offices and other dentists’ practices related to cardiovascular risk management.

## Material and Methods

A cross-sectional study was conducted with dentists in Colombia in 2022. Participants were invited to join voluntarily, following authorization for the use of information and acceptance of informed consent. This study was approved by the Ethics Committee of the University of Santander in Colombia.

The sample frame consisted of 1,442 individuals. The sample size was calculated using Epidat version 4.1 software, assuming that 75% of dentists would correctly identify risk factors for cardiovascular disease (CVD). (21) , a confidence level of 95%, and a precision of 5%. With these parameters, the sample size was 241 dentists. Simple random sampling was used to select the sample, however, after three weeks of sending the invitation via email, the response was poor (n= 67), so we decided to use the snowball sampling method and seek volunteers via social networks or by referral from those who had already responded. Hypothesis test of equality of proportions (Chi-square test) and means (two-sample t test) of the characteristics of the participants selected by simple random sampling and those who answered the survey by snowball sampling were tested, and no significant differences were found between the samples (*p*>0.05).

The survey was designed based on questions from two studies (17,21). The validity of the instrument was assessed with input from 10 thematic experts, all considered stakeholders. They held postgraduate degrees in periodontics, endodontics, microbiology, and research, each with at least 10 years of experience; eight of them were university professors. The validity estimate was assessed using Lawshe Content Validity Ratio (0.83) and internal consistency was assessed using the Cronbach Alpha (0.77). The first part of the survey included sociodemographic variables such as sex, age, and educational level, as well as variables related to dental practice, including hours of practice, type of practice (individual or group -shared with other colleagues or professionals-), type of institution (private or public), and availability of support staff. The second part of the survey assessed knowledge, attitudes, and practices related to systemic health issues. A Google Forms was created to collect information, it included informed consent, participants could access the complete survey once they expressed consent.

To investigate BPM in dental offices, the survey included the questions: “Regarding the evaluation of systemic health, do you take blood pressure?”, “How often do you take blood pressure?”, and “Do you use an automatic blood pressure measuring device?” Regarding practices, questions assessed whether modifiable risk factors were addressed or discussed, with response options of “Yes” or “No”. Questions also inquired about the likelihood of performing practices related to glucose measurements in dental offices, referring patients for laboratory tests or to primary care physicians, communicating with doctors, and providing counseling and education about risk factors. Response options were “very likely,” “probable,” “somewhat likely,” or “not at all likely,” which were grouped to dichotomize the variable.

Statistics and data analysis: Data were expressed as frequency (%) for categorical variables and means (SD) for continuous variables. Practices related to cardiovascular risk management were compared based on whether blood pressure was measured or not, using Chi-square analysis. Logistic regression analysis was used to examine the association between poor practices and the lack of blood pressure measurement in dental offices. Model 1 represents the crude analysis, while Model 2 includes adjustments for age, education, and support staff. The level of significance was set at 0.05. All analyses were performed using Stata Statistical Software (17.0 BE-Basic Edition; StataCorp, Texas, USA).

## Results

 Table 1 shows the general characteristics of the dentists interviewed (n= 232). Of those interviewed, 69.0% were women, 38.8% had no specialty or master’s degree, 73.3% reported performing their clinical practice individually, i.e., without other dentists or professionals, 86.6% worked in private institutions, approximately 74.1% had an assistant or hygienist to help them in their clinical practice, and they worked an average of 7.3 ±1.9 hours per day.

**Figure 1:**
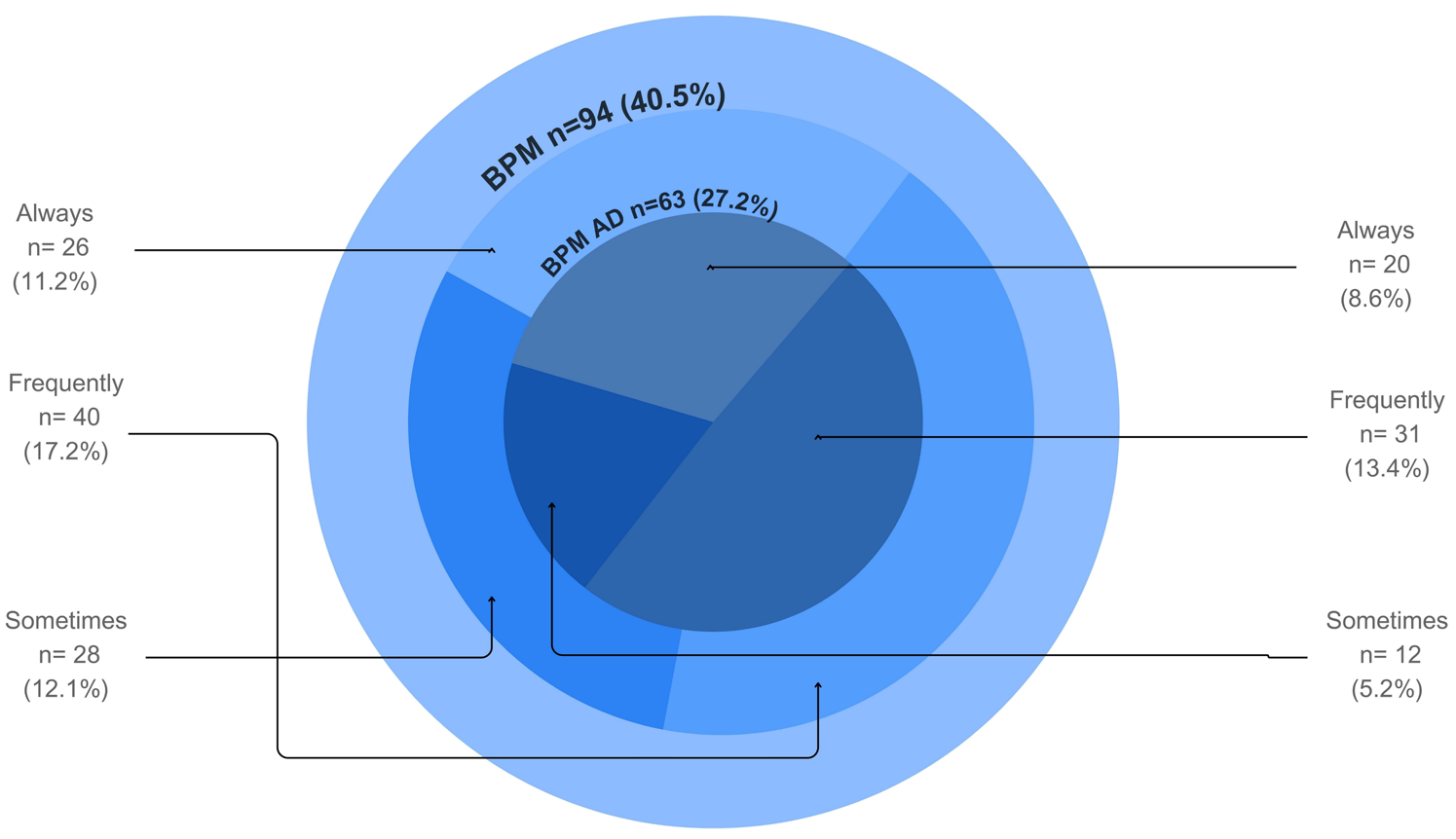
Distribution of blood pressure measurements in the dental office. BPM: Blood pressure measurement. AD: Automatic devices.

Figure 1 shows that 40.5% of the dentists measured blood pressure in the dental office, with 27.2% using an automatic device. In summary, 8.6% of the dentists measured blood pressure with an automatic device during every patient visit.

 Table 2 shows the differences in proportions of practices related to cardiovascular risk management and BPM in the dental office. In general, dentists inquire about risk factors; however, among those who did not measure blood pressure, the highest proportion were those who did not ask about tobacco use (80.9%; *p*= 0.036), followed by dyslipidemias (72.7%; *p*= 0.001), alcohol consumption (72.6%; *p*= 0.014), obesity (69.9%; *p*= 0.029), and physical inactivity (67.3%; *p*= 0.025).

 Table 3 shows the likelihood reported by dentists of engaging in practices related to patient referral, evaluation, counseling, and education about risk factors, with these findings complemented by  Table 4. A greater number of dentists reported that they would probably perform all the actions proposed. However, the differences between those who reported not doing so were more pronounced in relation to whether or not Adjusting for age, education and support staff, an association was observed between the absence of practices related to inquiring about risk factors such as alcohol use (OR 2.61 CI 95% 1.34-5.08), physical inactivity (OR 2.03 CI 95% 1.16-3.53), dyslipidemias (OR 2.93 CI 95% 1.60-5.37) and obesity (OR 2.24 CI 95% 1.20-4.18), and not taking blood pressure in the dental office ( Table 4). Among the dentists who were unlikely to measure glucose in the dental office, 66.9% (*p*= 0.013) did not take blood pressure (OR 1.79 CI 95% 1.04-3.09). Additionally, 79.7% (*p*= <0.001) of those who did not refer patients to a laboratory for glucose measurement also reported not taking blood pressure (OR 3.83 CI 95% 1.95-7.54). Also significant was the association between not calling a medical professional to arrange for a patient’s treatment and not taking blood pressure (OR 2.69 CI 95% 1.44-5.05), as well as not educating people with obesity about the risk of systemic disease (OR 1.85 CI 95% 1.02-3.37).

## Discussion

The present study established that there is an association between lack of blood pressure measurement in the dental office and the omission of other practices related to cardiovascular risk management by the dentist. Of the dentists surveyed, 59.5% did not measure blood pressure in the dental office. Among those who did, 27.2% used automatic equipment, and only 8.6% measured blood pressure for every patient who visited the dental office. In addition to the low proportion of automatic equipment use, it is important to emphasize that measuring blood pressure with validated devices is a key component of adopting best practices for the prevention and control of cardiovascular diseases (12,13,22).

Our findings align with other studies, which highlight that while dentists routinely inquire about their patients’ medical history and express willingness to incorporate cardiovascular risk management practices, only one in four actually measures blood pressure, regardless of the patient’s condition (17). The routine measurement of blood pressure in dental offices is notably low, with studies reporting proportions ranging from 1.3% and 25% (17,18,20,23). Our study found that only 11.2% of dentists consistently measure blood pressure, regardless of the device used. We did not specifically inquire about who measures blood pressure; however, we found that 74.1% of dentists have support staff, which could facilitate blood pressure measurement. A survey indicates that most hygienists recognize the importance of identifying patients who could benefit from interventions to prevent or control medical conditions and perform early detection of hypertension, diabetes, CVD, and other infectious diseases such as hepatitis and HIV. Hygienists are also willing to conduct screenings that provide immediate results, discuss these results with patients, and refer them for medical consultations (24).

Dentists can play a crucial role in detecting and managing hypertension during dental visits. Referring patients to a physician can significantly reduce the morbidity and mortality associated with hypertension (17,25-27). In this regard, our findings present a moderately positive picture: 85.3% of the dentists surveyed indicated that they were likely to refer patients to a physician for follow-up on signs and symptoms detected during the dental consultation. However, it is noTable that not all of these dentists measured blood pressure (58.1%). On the other hand, it is necessary to strengthen communication with physicians. Although 68.5% of dentists indicated that they were likely to communicate with physicians to arrange treatment, it was found that not communicating with medical professionals was associated with not measuring blood pressure during the dental visit. The literature suggests that dentists who include such practices often adopt a holistic perspective on patient health, viewing their roles as extending beyond the oral cavity to encompass broader health concerns (28).

The potential public health impact of integrating the dental office into primary care has been studied in relation to cardiovascular risk factors such as diabetes mellitus and smoking (29). In general, the dentists who participated in our study inquired about cardiovascular risk factors, with the highest rate for tobacco use (90.5%) and the lowest for physical inactivity (53.9%). We also found that around 45% of the dentists who measured blood pressure also asked about tobacco use, alcohol consumption, physical inactivity, diet, dyslipidemia, and excess weight. However, omitting to evaluate or discuss cardiovascular risk factors with patients was associated with not taking blood pressure in the dental office in all cases, except for tobacco use. It has been identified that dentists who screen for obesity and hypertension are also more likely to agree that screening for cardiovascular disease should be part of the dentist’s role as health professionals (28). Improving dentist involvement in cardiovascular risk management is likely to require patient availability. A survey on patient attitudes toward in-office testing revealed that most patients view it as important for dentists to screen for conditions like diabetes and hypertension. They are generally willing to undergo screening tests and discuss results with the dental team (29,30).

The findings of this research highlight the need to strengthen the competencies of dentists in cardiovascular risk management. We concur that enhancing these skills could facilitate their involvement in managing cardiovascular risk (28). A previous publication using the same sample of dentists revealed gaps in identifying risk factors with established evidence for cardiovascular disease development, such as dietary practices, hypertension, dyslipidemia, and obesity (31). In our reflection, we believe that enhancing dentists’ competencies will bolster their confidence and effectiveness in managing patients at risk of CVD. Literature indicates that around 79.5% of dentists perceive managing patients with hypertension as risky and acknowledge a gap in knowledge and attitudes towards proper hypertension management within the dental profession (18,32). However, it is encouraging that a significant proportion of dentists recognize the value of education on hypertension and cardiovascular risk (20,21,28). Complementarily, inadequate measurement undermines the potential benefits of detecting hypertension outside of a clinical or physician’s office setting. A review suggests that poor blood pressure assessment is more common among NPHWs, such as dentists and optometrists, compared to physicians. However, there is currently no evidence to confirm or refute this hypothesis (33). It is important to highlight that there is no evidence regarding the specific practices of BPM in dental offices. The issue may not lie with the use of automatic devices themselves, but rather with errors induced by the patient’s posture, which may not be controlled or avoided by the professional (34). In any case, the call to involve NPHWs in cardiovascular risk management is increasingly strategic, especially in detection tasks such as BPM and health education on issues such as counseling for the abandonment of harmful habits and the adoption of healthy lifestyles (14).

This study highlights as strengths its findings that support enhancing dentists’ competencies and active role in detecting and managing hypertension, evaluating and counseling on other cardiovascular risk factors, and improving referral processes with physicians and other health professionals. The research is noted as the first of its kind in Latin America, making it valuable for the push towards multidisciplinary teams in countries like Colombia undergoing health system changes. However, potential limitations are acknowledged, including the self-administered nature of the questionnaires, which requires cautious interpretation of results and prevents generalization. Additionally, the snowball sampling method may have introduced bias by primarily recruiting participants already interested in the topic. Another limitation is that the multivariate analysis did not control for potential confounding variables.

## Conclusions

A low proportion of dentists measure blood pressure in the dental office with automatic devices, reflecting poor adherence to evidence-based clinical practices. Consequently, opportunities for referring individuals for hypertension diagnosis or blood pressure management are often missed.

Although most dentists report inquiring about or assessing modifiable risk factors, making referrals, providing counseling, and offering education, the lack of BPM in dental offices is linked to the omission of other risk management practices. The practices that are omitted or less likely to be performed include inquiring about or assessing alcohol use, physical inactivity, high cholesterol, and obesity; performing glucose measurements in the dental office; referring patients to a laboratory for glucose measurement; contacting medical professionals for patient treatment; and educating patients with obesity.

Consequently, there is a need to strengthen dentists’ practices comprehensively and promote their integration into multidisciplinary teams involved in the prevention, detection, and control of hypertension. The findings of this research suggest the urgent need for implementing health education actions in both academic and professional settings to promote evidence-based, collaborative, and effective dental practices.

## Figures and Tables

**Table 1:** Participants’ characteristics.

Characteristics	Blood pressure measurement		Total (n= 232)
	Yes n= 94	No n= 138	
Age -mean (SD)	38.4 (12.2)	40.8 (11.8)	39.8 (12.0)
Sex			
Female	59 (62.8)	101 (73.2)	160 (69.0)
Male	35 (37.2)	37 (26.8)	72 (31.0)
Education -n (%)			
Professional degree	32 (34.0)	58 (42.0)	90 (38.8)
Clinical specialty	36 (38.3)	54 (39.1)	90 (38.8)
Other specialty	19 (20.2)	22 (15.9)	41 (17.8)
Master	7 (7.5)	4 (2.9)	11 (4.7)
Practice type -n (%)			
Group	29 (30.8)	33 (23.9)	62 (26.7)
Individual	65 (69.2)	105 (76.1)	170 (73.3)
Institution type -n (%)			
Public	9 (9.6)	22 (15.9)	31 (13.4)
Private	85 (90.4)	116 (84.1)	201 (86.6)
Support staff -n (%)			
Assistant or dental hygienist	63 (67.0)	109 (79.0)	172 (74.1)
No support	31 (33.0)	29 (21.0)	60 (25.9)
Hours of care practice -mean (SD)	7.2 (1.9)	7.4 (1.9)	7.3 (1.9)

**Table 2:** Cardiovascular risk factors for which dentists assess or discuss with their patients.

Practices	Blood pressure measurement		p value
	Yes	No	
Inquires about tobacco use			0.036
Yes	90 (42.7)	121 (57.3)	
No	4 (19.1)	17 (80.9)	
Inquires about alcohol consumption			0.014
Yes	77 (45.3)	93 (54.7)	
No	17 (27.4)	45 (72.6)	
Inquires about physical inactivity			0.025
Yes	59 (47.2)	66 (52.8)	
No	35 (32.7)	72 (67.3)	
Inquire about diet			0.440
Yes	68 (39.1)	106 (60.9)	
No	26 (44.8)	32 (55.2)	
Inquires about dyslipidemias			0.001
Yes	70 (48.6)	74 (51.4)	
No	24 (27.3)	64 (72.7)	
Inquires about obesity			0.029
Yes	72 (45.3)	87 (54.7)	
No	22 (30.1)	51 (69.9)	

**Table 3:** Likelihood to perform practices related to patient referral, evaluation, counselling and education about risk factors.

Practices	Blood pressure measurement		p value
	Yes	No	
Likely to refer patients to a physician to follow up on signs and symptoms detected during the dental appointment			0.294
Yes	83 (41.9)	115 (58.1)	
No	11 (32.4)	23 (67.6)	
Likely to contact a medical professional to coordinate treatment			0.002
Yes	75 (47.2)	84 (52.8)	
No	19 (26.0)	54 (74.0)	
Likely to refer patients to the lab for glucose measurement			<0.001
Yes	80 (49.1)	83 (50.9)	
No	14 (20.3)	55 (79.7)	
Likely to assess patients using portable glucose meter			0.013
Yes	53 (49.1)	55 (50.9)	
No	41 (33.1)	83 (66.9)	
Likely to offer tobacco cessation counseling			0.289
Yes	61 (43.3)	80 (56.7)	
No	33 (36.3)	58 (63.7)	
Likely to educate patients with obesity about systemic disease risk			0.086
Yes	70 (44.3)	88 (55.7)	
No	24 (32.4)	50 (67.6)	
Likely to educate patients about the association between glycemic control and dental complications			0.143
Yes	76 (43.2)	100 (56.8)	
No	18 (32.1)	38 (67.9)	

**Table 4:** Association between the lack of blood pressure measurement in the dental office and dentists´ practices related cardiovascular risk management.

Practices	Model 1			Model 2		
	OR	CI 95%		OR	CI 95%	
Does not inquire about tobacco use	3.16	1.03	9.72	3.08	0.99	9.62
Does not inquire about alcohol consumption	2.19	1.16	4.13	2.61	1.34	5.08
Does not inquire about physical inactivity	1.84	1.08	3.14	2.03	1.16	3.53
Does not inquire about diet	0.79	0.43	1.44	0.88	0.47	1.63
Does not inquire about dyslipidemias	2.52	1.42	4.47	2.93	1.60	5.37
Does not inquire about obesity	1.92	1.06	3.46	2.24	1.20	4.18
Likely not to assess patients using portable glucose meter	1.95	1.15	3.32	1.79	1.04	3.09
Likely not to refer patients to the lab for glucose measurement	3.79	1.95	7.34	3.83	1.95	7.54
Likely not to refer patients to a physician to follow up on signs and symptoms detected during the dental appointment	1.51	0.70	3.27	1.70	0.77	3.76
Likely not to contact a medical professional to coordinate treatment	2.54	1.38	4.66	2.69	1.44	5.05
Likely not to offer tobacco cessation counseling	1.34	0.78	2.30	1.44	0.82	2.52
Likely not to educate patients with obesity about systemic disease risk	1.66	0.93	2.96	1.85	1.02	3.37
Likely not to educate patients about the association between glycemic control and dental complications	1.60	0.85	3.03	1.66	0.86	3.18

## Data Availability

The datasets used and/or analyzed during the current study are available from the corresponding author.
